# Identification of the origin of tumor in vein: comparison between CEUS LI-RADS v2017 and v2016 for patients at high risk

**DOI:** 10.1186/s12880-022-00912-4

**Published:** 2022-10-29

**Authors:** Wen-juan Tong, Mei-qing Cheng, Man-xia Lin, Hang-tong Hu, Jia-min Pan, Hui Huang, Ying Wang, Xiao-yan Xie, Ming-de Lu, Ming Kuang, Yang Huang, Wei Wang

**Affiliations:** 1 Department of Medical Ultrasonics, Institute of Diagnostic and Interventional Ultrasound, The First Affiliated Hospital, Sun Yat-Sen University, Guangzhou, People’s Republic of China; 2 Department of Hepatobiliary Surgery, The First Affiliated Hospital, Sun Yat-Sen University, Guangzhou, People’s Republic of China; 3 Department of Medical Ultrasound, The First Affiliated Hospital of Guangzhou Medical University, Guangzhou, People’s Republic of China

**Keywords:** Ultrasonography, Hepatocellular carcinoma, Intrahepatic cholangiocarcinoma, Contrast media

## Abstract

**Objectives:**

To compare the diagnostic performance of the Contrast-Enhanced Ultrasound (CEUS) Liver Imaging Report and Data System (LI-RADS) v2016 and v2017 in identifying the origin of tumor in vein (TIV).

**Methods:**

From April 2014 to December 2018, focal liver lesions (FLLs) accompanied by TIV formation in patients at high risk for hepatocellular carcinoma (HCC) were enrolled. Histologic evaluation or composite imaging reference standard were served as the reference standard. Each case was categorized according to the CEUS LI-RADS v2016 and v2017, respectively. Diagnostic performance of CEUS LI-RADS v2016 and v2017 in identifying the originated tumor of TIV was validated via sensitivity, specificity, accuracy, positive predictive value (PPV) and negative predictive value.

**Results:**

A total of 273 FLLs with TIV were analyzed finally, including 266 HCCs and 7 non-HCCs. In v2016, when adopting all TIV as LR-5V, the accuracy and PPV in identifying the originated tumor were both 97.4%. In v2017, when assigning TIV according to contiguous FLLs CEUS LI-RADS category, the accuracy and PPV were 61.9% and 99.4% in subclass of LR-5 as the diagnostic criteria of HCC, and 64.1% and 99.4% in subclass of LR-4/5 as the criteria of HCC diagnosis. There were significant differences in diagnostic accuracy between CEUS LI-RADS v2016 and v2017 in identifying the originated tumor of TIV (*p* < 0.001).

**Conclusions:**

CEUS LI-RADS v2016 could be better than v2017 in identifying the originated tumor of TIV.

**Supplementary Information:**

The online version contains supplementary material available at 10.1186/s12880-022-00912-4.

## Introduction

Primary liver cancer (PLC) is the fifth most common tumor worldwide and the second most frequent cause of cancer-related mortality [1], and vascular invasion is an important risk factor determining the outcome. The portal vein tumor thrombosis (PVTT) is the common type of tumor in vein (TIV), and occurs most frequently in hepatocellular carcinoma (HCC) patients [2, 3]. PVTT is devastating complication of advanced PLC and has been regarded as unsuitable or poorly suitable for operation. Different types of PLC have different treatment regimens, and medical therapy is the most commonly used treatment for advanced PLC. In the past 10 years, sorafenib has been the first-line systemic drug for advanced HCC [4]. Recently, atezolizumab plus bevacizumab has become the first and only FDA-approved immunotherapy for first-line treatment of unresectable HCC, which resulted in better overall and progression­free survival outcomes than sorafenib [5]. Gemcitabine plus platinum-based has been widely used as the standard chemotherapy for unresectable or metastatic intrahepatic cholangiocarcinoma (ICC) [6], and various immunotherapeutic drugs are still being studied in clinical trials and no definite conclusions have been drawn yet [7]. Hence, clarifying the originated tumor of TIV is helpful for clinical treatment decision-making.

In clinical practice, patients with suspected HCC and vascular invasion usually undergo contrast-enhanced ultrasound (CEUS), computed tomography (CT) or magnetic resonance imaging (MRI) to get the assessment. Previous studies showed that dual-energy CT with iodine quantification, three-dimensional reconstruction of multiple- slice CT, and gadoxetic acid–enhanced MRI have a good diagnostic value for TIV [8, 9]. In addition, it has been reported that CEUS appeared to be significantly superior to CT for detection TIV [10]. TIV occurs in portal, hepatic vein or inferior vena cava, and most frequently in portal vein and its branches. Previous study has indicated that HCC account for a majority of TIV (70.9%) [11]. Besides, intratumoral vein is another form for intrahepatic vein invasion, which means that the intrahepatic vein is encompassed by the tumor without displacement and occlusion, and is a common way of vascular invasion in ICC [12–14].

The CEUS Liver Imaging Reporting and Data System (LI-RADS) defined “definite enhancing soft tissue in vein regardless of visualization of parenchymal mass or nodule” as LR-5V in v2016 while as LR-TIV in v2017 [15, 16]. The LR-TIV v2017 in CEUS LI-RADS replaced the LR-5V v2016. LR-5V v2016 regards originated tumor of macrovascular invasion and tumor thrombus in vein as HCC directly, and LR-TIV v2017 takes the originated tumor of TIV into consideration and LR-TIV subclasses are defined. In LR-TIV v2017, if contiguous Focal liver lesion (FLL) categorized as LR-5, the originated tumor of TIV is definitely HCC; if contiguous FLL categorized as LR-4 or it associated with infiltrative mass, the originated tumor of TIV is probably HCC; if contiguous FLL categorized as LR-M, the originated tumor of TIV may be due to non-HCC malignancy; otherwise, it is considered to etiology uncertain.

At present, most studies paid more attention to the diagnostic performance of CEUS LR-3/4/5 and LR-M [17–19], and there is no study focused on the comparison between LR-5V v2016 and LR-TIV v2017. Therefore, we aimed to explore the etiologic distribution of originated tumor of TIV, and to compare the diagnostic performance for identifying the originated tumor of TIV between CEUS LI-RADS v2016 and v2017.

## Materials and methods

Our retrospective study was approved by the institutional ethics committee, and written informed consent was obtained from each patient for CEUS examination.

### Study population

We reviewed our database of consecutive patients who underwent both baseline ultrasound (US) and CEUS between April 2014 and December 2018 at a single hospital. The inclusion criteria were (1) High risk of HCC according to CEUS LI-RADS guideline [15, 16]. (2) All FLLs were newly detected by US and CEUS. (3) All patients met the diagnosis of intrahepatic TIV reference standard, details are described below. The exclusion criteria were (1) Cirrhosis due to congenital hepatic fibrosis or vascular disorder such as Budd-Chiari syndrome, hereditary hemorrhagic telangiectasia, cardiac congestion, chronic portal vein occlusion, or diffuse nodular regenerative hyperplasia. (2) Poor image quality could not to categorize CEUS LI-RADS category.

### Reference standard

**Fig. 1:**
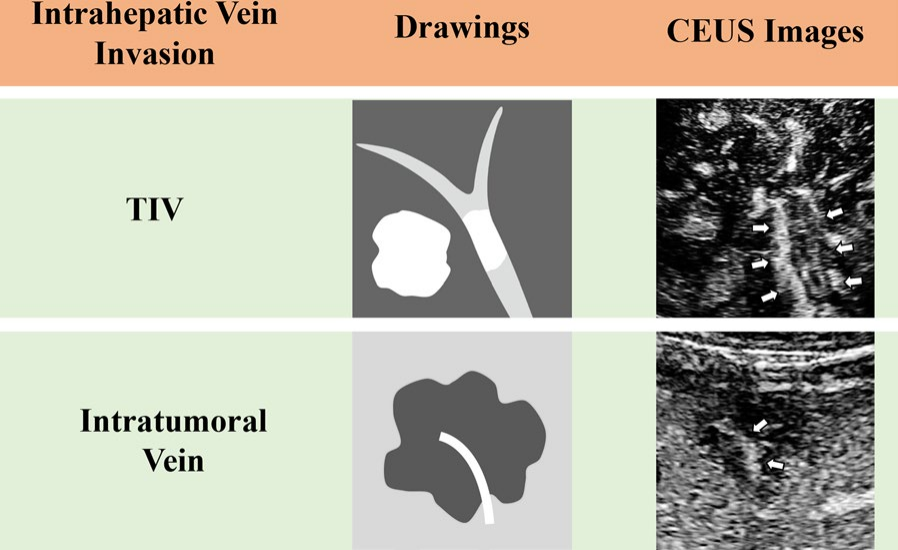
The way of intrahepatic vein invasion. CEUS: Contrast-Enhanced Ultrasound, TIV: Tumor in vein

The reference standard for TIV was definite enhancing soft tissue in vein, regardless of visualization of a parenchymal mass and visible adjacent primary tumor in liver parenchyma (Fig. 1) [15, 16], then with the surgical pathologic confirmation or three months imaging follow-up remained the diagnosis of TIV. All FLLs were diagnosed by either histopathology (surgery or puncture biopsy pathology, the biopsy procedure were described in Additional file 1: Section S1) or composite imaging reference standard. The composite standard for HCC was FLLs with CECT or MRI scans characterized by the arterial phase hyperenhancement (APHE) and washout appearance in the portal venous phase or delayed phase in a size greater than 1 cm in diameter [20]. Other malignant lesions were diagnosed by histopathology only.

### Imaging techniques

Ultrasound examinations were performed as follows: (1) Aplio 500 (Toshiba Medical Systems, Tokyo, Japan) scanner equipped with a 375BT convex transducer (frequency range, 1.9–6.0 MHz) and a Contrast Harmonic Imaging mode; and (2) Aixplorer Ultrasound system (SuperSonic Imagine, Aix-en-Provence, France) scanner equipped with the SC6-1 convex probe (frequency range, 1.0–6.0 MHz). Ultrasound examination was performed in a standardized fashion. First, all patients underwent unenhanced abdominal and hepatic sonography using the conventional US. The location, number, size and sonographic features of the FLLs and suspicious TIV were recorded. Select the most suspicious FLL for CEUS in case with more than one lesion. When the examination was done applying CEUS mode, low-mechanical index and dual screen format were used. 2.4 mL of the SonoVue was injected intravenously and immediately flushed with 5 mL 0.9% saline, then the FLL was observed for at least 5 min. The specific features appear was recorded, which could be used to differentiate the different liver masses. If a suspicious TIV was found in US or at first time of CEUS, then injecting 2.4 mL of the SonoVue again to observe if there is enhancement in the arterial phase. The enhancement phases were defined as follow: arterial phases, < 30 s after contrast injection; portal venous phase, 31 s-2 min after injection; and late phase, 2 min to approximately 4–6 min after injection.

Imaging archiving and scanning parameters of CT and MRI examinations were described in Additional file 1: Section S2.

### Image analysis

All US and CEUS images were anonymous, and independently evaluated by two skilled radiologists (Y.H. and M.X.L.) who had more than 8 years’ experience in CEUS. They were unaware of clinical and other imaging information of the patients. If the diagnostic results were inconsistent, another radiologist (W.W.) with at least 11 years of experience in liver CEUS assessed the images to reach a consensus.

CEUS characteristics of FLL were recorded: (1) maximum diameter of the target lesion; (2) enhancement level in the arterial/portal/late phase (hyper/iso-/hypo-); (3) enhancement patterns in the arterial phase (rim/homogeneous/inhomogeneous/others); (4) washout time (early washout within 60 s or not) [21]; (5) degree of washout (no washout/mild washout/marked washout [< 2 min]) [15]. The enhancement level in the arterial phase (hyper/iso-/hypo-) of TIV was recorded. TIV was categorized as both LR-5V v2016 and LR-TIV v2017. The FLLs were classified according to CEUS LI-RADS category. The TIV originated from these contiguous FLLs were assessed according to FLLs CEUS LI-RADS category, and categorized as LR-3 subclass/ LR-4 subclass/ LR-5 subclass /LR-M subclass of LR-TIV v2017. The LR-3 subclass meant LR-TIV, etiology uncertain; the LR-4 subclass meant LR-TIV, probably due to HCC; the LR-5 subclass meant LR-TIV, definitely due to HCC; the LR-M subclass meant LR-TIV, may be due to non-HCC malignancy.

### Statistical analysis

Statistics were calculated using SPSS Statistics software v22.0 program and Microsoft Excel 2019. LR-5V v2016 considers the originated tumor of TIV as HCC. LR-TIV v2017 considers the originated tumor of TIV based on the contiguous FLL CEUS LI-RADS category. LR-5 subclass and LR-4/5 subclass of LR-TIV v2017 were used as diagnosis criteria for HCC separately. Diagnostic performance of CEUS LI-RADS v2016 and v2017 in identifying the originated tumor of TIV was assessed with sensitivity, specificity, accuracy, positive predictive value (PPV) and negative predictive value (NPV). McNemar’s test and Fisher's test was applied for comparison the diagnostic performance between CEUS LI-RADS v2016 and v2017 in identifying the originated tumor of TIV.

## Results

### Patients, FLLs and TIV characteristics

**Fig. 2:**
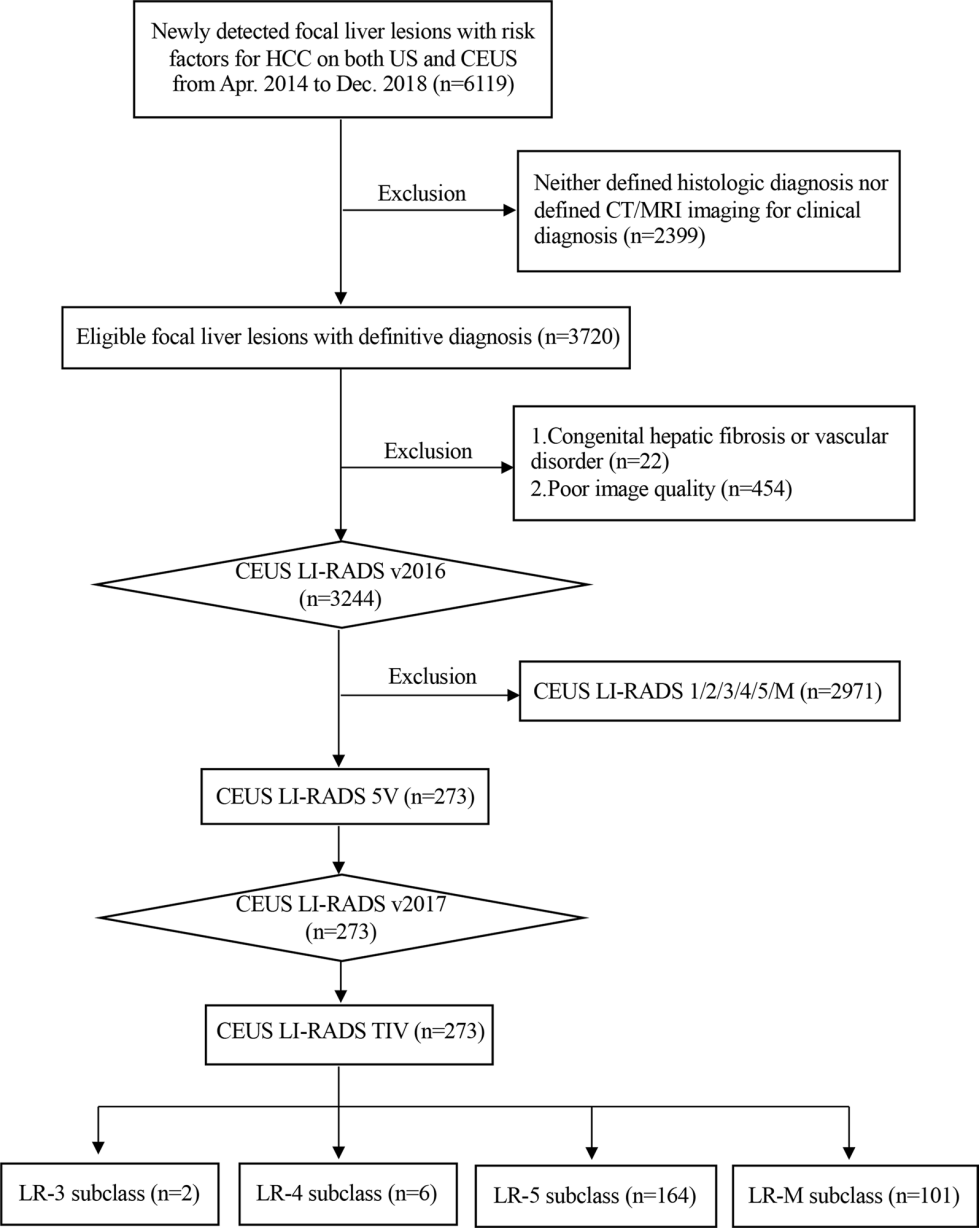
A flow diagram of our study sample. CEUS: Contrast-Enhanced Ultrasound, HCC: Hepatocellular Carcinoma, US: Ultrasound, LI-RADS: Liver Imaging Reporting and Data System

A flow diagram of our study is showed in Fig. 2. A total of 273 FLLs (mean size, 9.5 ± 3.71 cm) in 273 patients (median age, 50.0 years; 249 male) were final included. 168 (61.5%) of 273 FLLs developed in patients with chronic hepatitis B virus (HBV) infection only, 81 (29.7%) with chronic HBV infection and cirrhosis, 2 (0.7%) with chronic HCV infection and cirrhosis, 2 (0.7%) with chronic HBV and HCV infection and cirrhosis, and 20 (7.3%) with cirrhosis only. Patients and FLLs clinical characteristics are shown in Table 1. As for the reference standard, there were 266 HCC (97.4%) and 7 (2.6%) non-HCC malignancies. In 266 HCCs, 146 (54.9%) were diagnosed as HCC by pathology, and 120 (45.1%) were diagnosed as HCC by CECT or MRI images. In 7 non-HCC malignancies, including 3 ICCs (1.1%), 1 combined hepatocellular-cholangiocarcinoma (CHC) (0.4%), 1 hepatosarcoma (HSC) (0.4%), 1 primary neuroendocrine tumor (0.4%), and 1 other malignant tumor (0.4%), were confirmed by pathologic diagnosis. Other characters including US and CEUS characteristics, are presented in Additional file 1: Table S1.

**Table 1:** Patient and FLLs characteristics

Variable	Value
Patients (n = 273)	
No. of men	249 (91.2)
Median age (y)*	50 (22–68)
HCC risk factors	
Chronic HBV + Cirrhosis	81 (29.7)
Chronic HCV + Cirrhosis	2 (0.7)
Chronic HBV + Chronic HCV + Cirrhosis	2 (0.7)
Only Chronic HBV	168 (61.5)
Cirrhosis	20 (7.3)
Diagnosis	
HCC	266 (97.4)
ICC	3 (1.1)
CHC	1 (0.4)
Hepatosarcoma	1 (0.4)
Primary neuroendocrine tumor	1 (0.4)
Other malignant tumor	1 (0.4)
HCC diagnostic method (n = 266)	
Pathological confirmed	146 (54.9)
Composite imaging	120 (45.1)

Except where indicated, data are numbers of nodules and numbers in parentheses are percentages

*CHC* Combined hepatocellular-cholangiocarcinoma; *FLLs* Focal liver lesions; *HBV* Hepatitis B virus; *HCC* Hepatocellular Carcinoma; *HCV* Hepatitis C virus; *ICC* Intrahepatic cholangiocarcinoma

*Numbers in parentheses are the range of age

### Diagnostic performance of CEUS LI-RADS v2016 versus v2017

When applying CEUS LI-RADS v2017 (Table 2), in the 273 TIV cases, there were 2 (0.7%) LR-3 subclass of LR-TIV v2017 cases confirmed as HCC; 6 (2.2%) cases were LR-4 subclass and confirmed as HCC; 164 (60.1%) were LR-5 subclass including 163 HCCs and 1 HSC; 101 (37.0%) were LR-M subclass including 95 HCCs, 3 ICCs, 1 CHC, 1 primary neuroendocrine tumor, and 1 other malignant tumor. As the level of CEUS LI-RADS rose, the proportion of HCC in each level to the overall HCC increased gradually (LR-3 subclass, 0.8% [2/266]; LR-4 subclass, 2.3% [6/266]; LR-5 subclass, 61.3% [163/266]). In the 266 HCCs, 95 (35.7%) were categorized as LR-M subclass.

**Table 2:** CEUS LI-RADS v2017 categories of the originated tumor in TIV

Originated tumor	Subclass of LR-TIV			
	LR-3 subclass (n = 2)	LR-4subclass (n = 6)	LR-5 subclass (n = 164)	LR-M subclass (n = 101)
HCC (n = 266)	2 (100.00)	6 (100.0)	163 (99.4)	95 (94.1)
ICC (n = 3)	0	0	0	3 (3.0)
CHC (n = 1)	0	0	0	1 (1.0)
Hepatosarcoma (n = 1)	0	0	1 (0.6)	0
Neuroendocrine tumor (n = 1)	0	0	0	1 (1.0)
Other malignant tumor (n = 1)	0	0	0	1 (1.0)

Data in parentheses are percentages

*CEUS* Contrast-Enhanced Ultrasound; *CHC* Combined hepatocellular-cholangiocarcinoma; *HCC* Hepatocellular Carcinoma; *ICC* Intrahepatic cholangiocarcinoma; *LI-RADS* Liver Imaging Reporting and Data System; *TIV* Tumor in vein

In Table 3, when regarding LR-5 subclass of LR-TIV v2017 as a criteria of HCC diagnosis, the sensitivity, specificity, accuracy, PPV and NPV were 61.3%, 85.7%, 61.9%, 99.4% and 5.5%, respectively. when regarding LR-4/5 subclass of LR-TIV v2017 as a criteria of HCC diagnosis, the sensitivity, specificity, accuracy, PPV and NPV were 63.5%, 85.7%, 64.1%, 99.4% and 5.8%, respectively.

**Table 3:** Diagnostic performance of LR-5V v2016 and LR-TIV v2017 in identifying the originated tumor of TIV

CEUS LI-RADS category	Sensitivity	*p* value	Specificity	*p* value	Accuracy	*p* value	PPV	*p* value	NPV	*p* value
LR-5V (n = 273)	–	–	–	–	97.4 (96, 99) [266/273]	–	97.4 (96, 99) [266/273]	–	–	–
LR-5 subclass of LR-TIV (n = 164)	61.3 (55, 67) [163/266]	–	85.7 (51, 121) [6/7]	–	61.9 (56, 68) [169/273]	< 0.001	99.4 (98, 101) [163/164]	0.268	5.5 (1, 10) [6/109]	–
LR-4/5 subclass of LR-TIV (n = 170)	63.5 (58, 69) [169/266]	–	85.7 (51, 121) [6/7]	–	64.1 (58, 70) [175/273]	< 0.001	99.4 (98, 101) [169/170]	0.161	5.8 (1, 10) [6/103]	–

Data are percentages, data in parentheses are 95% confidence intervals, and data in brackets are numbers of cases

*CEUS* Contrast-Enhanced Ultrasound; *LI-RADS* Liver Imaging Reporting and Data System; *NPV* Negative predictive value; *PPV* Positive predictive value; *TIV* Tumor in vein

When applying CEUS LI-RADS v2016 (Table 3), all cases were categorized as LR-5V v2016 and considered the originated tumor as HCC, the accuracy and PPV were both 97.4%. Statistical analysis revealed that the PPV of LR-5V v2016 and LR-TIV v2017 were both pretty high without significant differences (*p* > 0.05, both LR-5 subclass and LR-4/5 subclass of LR-TIV v2017). The accuracy of LR-5V v2016 was higher than that of LR-TIV v2017 with remarkable differences (*p* < 0.001, both LR-5 subclass and LR-4/5 subclass of LR-TIV v2017).

### LR-5 and LR-M subclasses of LR-TIV v2017

**Fig. 3:**
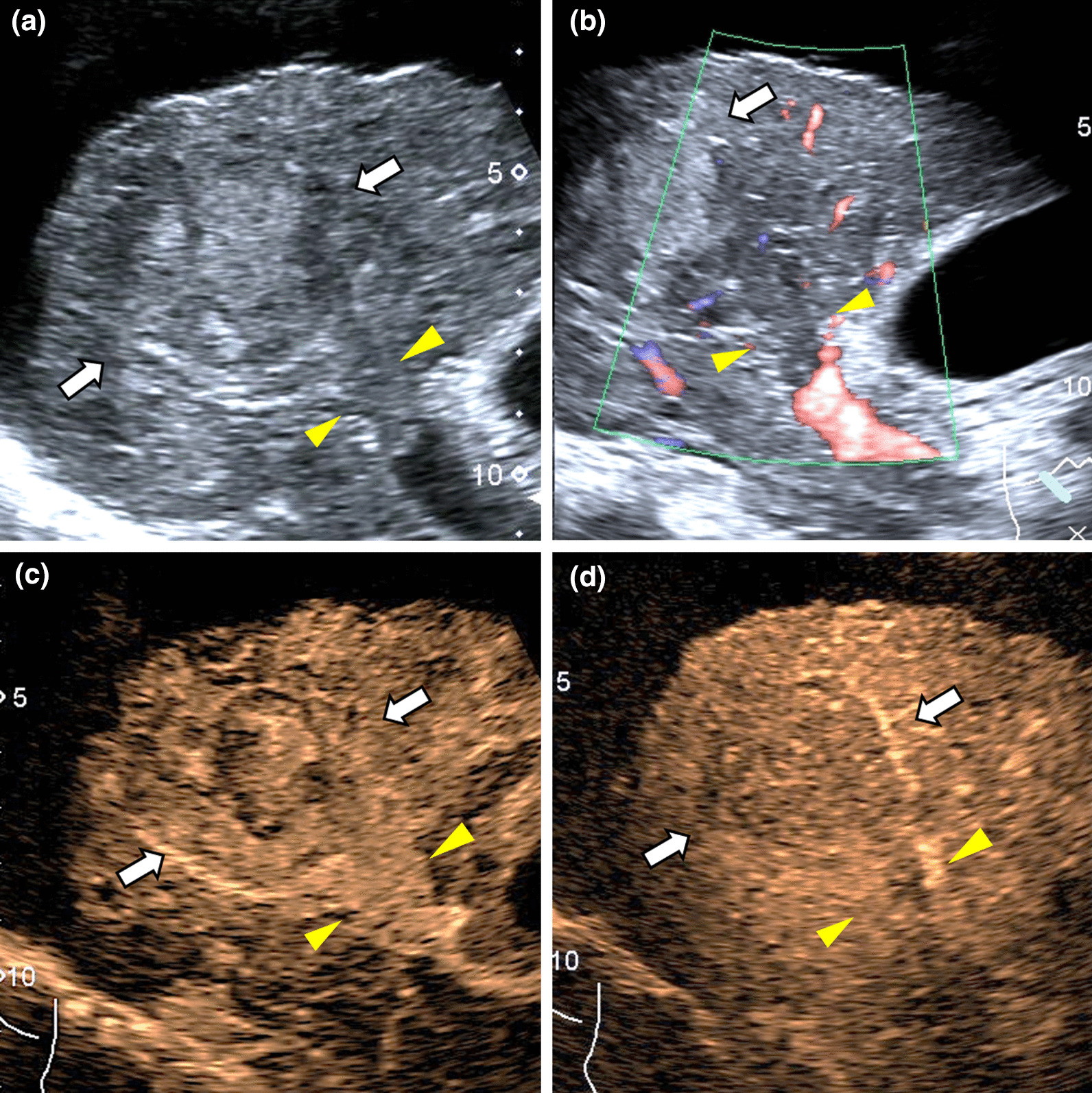
It illustrates an HCC lesion categorized LR-5 subclass of LR-TIV. A 4.0 cm HCC lesion in a 67-year-old man with chronic HBV infection and cirrhosis. (**a**) The heterogenous lesion (white arrowheads) with portal vein tumor thrombosis (yellow triangle) were demonstrated at B-mode ultrasound. (**b**) Color Doppler US showed few doted-like flows in the tumor thrombosis. (**c**) CEUS showed the lesion and tumor thrombosis displayed APHE at 23 s after SonoVue injection. (**d**) Mild washout was presented in the delayed phase (134 s). The lesion was proved to be low-grade HCC (G3) on pathologic analysis. APHE: Arterial phase hyperenhancement, CEUS: Contrast-Enhanced Ultrasound, HBV: Hepatitis B virus, HCC: Hepatocellular Carcinoma, US: Ultrasound

In Table 2, 164 (60.1%) TIV were categorized as LR-5 subclass of LR-TIV v2017 including 163 (99.4%) HCCs (Fig. 3) and 1 (0.6%) HSC. The FLL of HSC showed inhomogeneous hyperenhancement in the arterial phase, no marked washout (< 2 min), and washout time > 60 s.

**Fig. 4:**
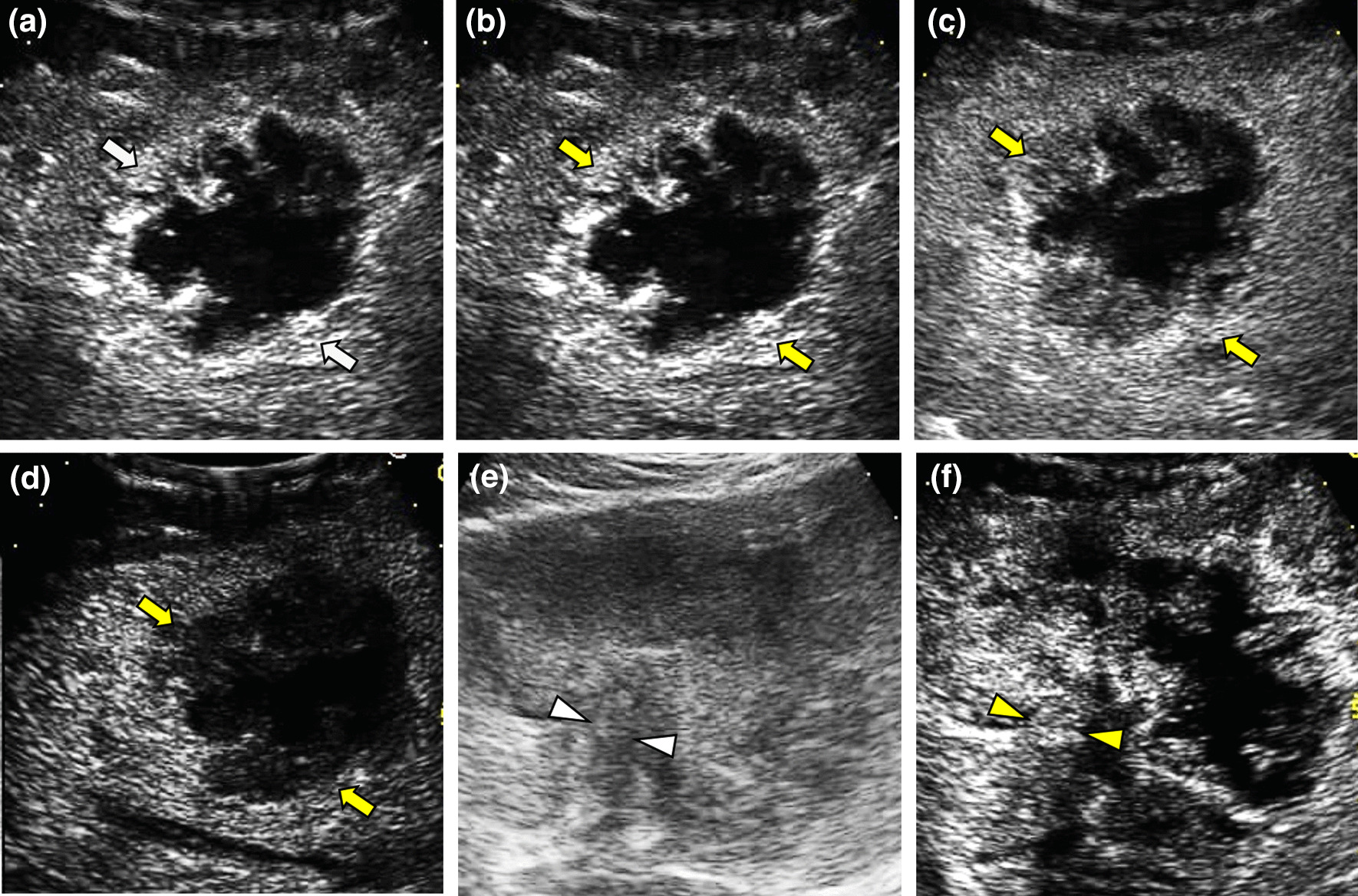
It illustrates an HCC lesion categorized LR-M subclass of LR-TIV. A 10.8 cm HCC lesion in a 67-year-old man with chronic HBV infection and cirrhosis. (**a**) The heterogenous lesion (white arrowheads) were demonstrated at B-mode ultrasound. (**b**) Rim hyperenhancement was presented (yellow arrowheads) at 23 s after SonoVue injection. (**c**) Mild washout was presented in the portal phase (32 s). (**d**) Then followed by marked washout at 112 s. (**e**) TIV closed to FLL was presented (white triangle) at B-mode ultrasound. (**f**) CEUS showed the TIV displayed APHE at 27 s after SonoVue injection (yellow triangle). APHE: Arterial phase hyperenhancement, CEUS: Contrast-Enhanced Ultrasound, FLLs: Focal liver lesions, HCC: Hepatocellular Carcinoma, US: Ultrasound, LI-RADS: Liver Imaging Reporting and Data System, TIV: Tumor in vein

Of the 101 LR-M subclass of TIV v2017, 95 (94.1%) were confirmed as HCCs, 3 were ICCs (3.0%), 1 was CHC (1.0%), 1 was primary neuroendocrine tumor (1.0%), and 1 was unspecified malignant tumor (1.0%). Among the 95 HCC categorized as LR-M, one (1.1%) FLL exhibited rim APHE, 91 (95.8%) FLLs exhibited washout time < 60 s, and 19 (20.0%) FLLs exhibited marked washout (< 2 min). Of those, there were 14 (14.7%) FLLs had two CEUS features and one (1.1%) FLL had all three CEUS features mentioned above (Fig. 4).

## Discussion

Until now, some studies just only mentioned LR-5V v2016 or LR-TIV v2017 in LIRADS categories without evaluating for the originated tumor of TIV [22–28]. No study focused on the comparison of diagnostic performance for identifying the originated tumor of TIV between CEUS LI-RADS v2016 and v2017, and our study was the first time to investigate this area. Our results demonstrated that the PPV of CEUS LI-RADS v2016 and v2017 in identifying HCC as originated tumor of TIV were both very high, and CEUS LI-RADS v2017 were slightly higher than CEUS LI-RADS v2016 without significant differences (*p* > 0.05). However, the diagnostic accuracy of CEUS LI-RADS v2016 in identifying the originated tumor of TIV was remarkable higher than that of v2017 (*p* < 0.001). Hence, we considered that CEUS LI-RADS v2016 could be better than CEUS LI-RADS v2017 in identifying the originated tumor of TIV.

In previous studies, HCC is the most common malignant lesion in high-risk background and the most important cause of TIV, and the percentages of TIV originating from HCC was approximately 55.6–100.0% in prior studies [23, 25–28]. Consistent with prior studies, the vast majority of FLLs accompanied by TIV were diagnosed as HCC according to diagnostic criteria in this study. According to CEUS LI-RADS v2016, LR-5V v2016 regarded all originated tumor of TIV as HCC, therefore it had a high accuracy in identifying originated tumor of TIV in this study. According to CEUS LI-RADS v2017, about one-third of TIV were categorized as LR-M subclass of LR-TIV v2017, which considered originated tumor of TIV may be due to non-HCC malignancy, and reduced diagnostic accuracy in identifying originated tumor of TIV. Hence, we considered that it was not necessary to identify the originated tumor of TIV via the contiguous FLLs subclass of LR-TIV v2017 category, since the process was complicated and the accuracy of LR-TIV v2017 to achieve it was not satisfied as well. And LR-5V v2016 was simple, intuitive and more accurate in identifying the origin of TIV.

Few studies focus on ICC with TIV. It was reported that the incidence of ICC in TIV is 8.7% [11]. In our study, there are only 3 ICCs in 273 TIV cases (1.1%), the pretty lower incidence is inconsistent with past previous studies but it is accordance with our working experience. TIV is the most common vascular invasion way of HCC [29], which definite enhancing soft tissue in vein regardless of visualization of parenchymal mass or nodule. And intratumoral vein is more frequent than TIV in ICC. Various progression patterns along the Glissonean sheath (sinusoidal space, vascular, lymphatic, perineural etc.) are observed, and tumor encompassing the vein without displacement and occlusion are more common [12–14]. In this study, we consider that TIV is only representative of intravascular tumor thrombus, not including the vein encompassed by the tumor without displacement and occlusion. For now, the difference in incidence of TIV in ICC between different studies may be the result of no clear distinction between TIV and intratumoral vein.

In this study, most of TIV were categorized as LR-5 subclass and the PPV of diagnosing HCC was 99.4%. Similar results were reported in previous studies that PPV ranged from 92.9 to 98.5% in LR-5 category [17, 23, 24, 30]. In addition, 37.0% of HCCs were categorized as LR-M subclass in our study. In previous studies, this proportion was about 14% [19, 30] with cases not separating from TIV alone and 4.8% [17] with cases excluding TIV completely, these proportion were obviously less than our result. With higher malignancy degree, the risk of portal vein infiltration is elevating [31], and the onset of washout is getting earlier in CEUS [32]. Huang JY et al. [30] mentioned that early washout (< 60 s) was more common in poorly differentiated HCCs or nonhepatocellular malignancies. Hence, we consider all FLLs accompanied by TIV may have relatively high malignancy degree and get earlier in inset of washout (< 60 s), which led more HCCs to be classified as LR-M in our study.

Previous study indicated CT/MRI achieved a high specificity (99.7%) while a relatively low sensitivity (67.3%) on TIV diagnosis according to Liver Imaging Reporting and Data System (LI-RADS) [8]. This relatively low sensitivity may resulted in the missed diagnosis of TIV, which influenced treatment selection and prognosis. It reports that CEUS demonstrated higher probability for detecting TIV than CT/MRI with its high temporal resolution which can detect arterial enhancement in real-time [10]. However, the operator dependency, depth dependency and image artifacts of CEUS may hindered the accurate detection of TIV, unlike CT and MRI. In contrast with CT and MRI, CEUS is a fast, effective, cheap and well tolerated technique, and can be performed bedside. Therefore, CEUS is a very reliable technique, CT and MRI should only be indicated in undetermined cases at CEUS [33].

Our study has several limitations. Firstly, it was a retrospective study, selection bias and missing data remained some potential problems. Secondly, the types of lesions were limited. In this study, HCC accounted for the vast majority of the sample, and only 7 cases were non-HCCs. Thirdly, our study is a single center study and has limited representativeness. A multicenter research is necessary to validate the applicability of CEUS LI-RADS v2016 and CEUS LI-RADS v2017 in identifying the originated tumor of TIV. Fourthly, the study was based on a Chinese population, and additional studies on different ethnic backgrounds are necessary to further validate and extrapolate to non-Chinese backgrounds.

## Conclusions

In conclusion, HCC is the most important cause of TIV, and there is significant difference between CEUS LI-RADS v2016 and CEUS LI-RADS v2017 in identifying the originated tumor of TIV. Although both of them have very high PPV, the accuracy of CEUS LI-RADS v2016 is remarkable higher than CEUS LI-RADS v2017, that means CEUS LI-RADS v2016 could be better than CEUS LI-RADS v2017 in identifying the originated tumor of TIV.

## Supplementary Information

**Additional file 1. Section S1:** The specific process of Focal liver lesion puncture biopsy procedure. **Section S2**: Image archiving and scanning parameters of CT and MRI. **Table S1**: FLLs with TIV images characteristics. Except where indicated, data are numbers of nodules or TIV cases and numbers in parentheses are percentages. * datas are Mean ± SD. / means NA. AP: Arterial phase; APHE: Arterial phase hyperenhancement; CEUS: Contrast-enhanced ultrasound; FLLs: Focal liver lesions; HCC: Hepatocellular Carcinoma; TIV: Tumor in vein; US: Ultrasound.

## Data Availability

The datasets used and analysed during the current study are available from the corresponding author on reasonable request.

## Abbreviations

APHE: Arterial phase hyperenhancement
CEUS: Contrast-enhanced ultrasound
CHC: Combined hepatocellular-cholangiocarcinoma
FLL: Focal liver lesion
HCC: Hepatocellular carcinoma
HSC: Hepatosarcoma
ICC: Intrahepatic cholangiocarcinoma
LI-RADS: Liver imaging reporting and data system
NPV: Negative predictive value
PLC: Primary liver cancer
PPV: Positive predictive value
PVTT: Portal vein tumor thrombosis
TIV: Tumor in vein
US: Ultrasound
