# Supplementary material for: Identification of the origin of tumor in vein: comparison between CEUS LI-RADS v2017 and v2016 for patients at high risk
Source: BMC Med Imaging. 2022 Oct 29;22:186. doi: 10.1186/s12880-022-00912-4 (PMC9617430; doi:10.1186/s12880-022-00912-4)
Supplement: Supplementary file 1 — Additional file 1. Section S1: The specific process of Focal liver lesion puncture biopsy procedure. Section S2: Image archiving and scanning parameters of CT and MRI. Table S1: FLLs with TIV images characteristics. Except where indicated, data are numbers of nodules or TIV cases and numbers in parentheses are percentages. * datas are Mean ± SD. / means NA. AP: Arterial phase; APHE: Arterial phase hyperenhancement; CEUS: Contrast-enhanced ultrasound; FLLs: Focal liver lesions; HCC: Hepatocellular Carcinoma; TIV: Tumor in vein; US: Ultrasound. [file 12880_2022_912_MOESM1_ESM.docx]

**Supplementary Material S1**

**Image archiving and scanning parameters of CT and MRI**

*CT examination*

Contrast-enhanced CT scans were performed using multidetector CT technology: a 64-detector-row (Aquilion CXL, Toshiba Medical, Tokyo, Japan) or a 320-detector-row CT machine (Aquilion One, Toshiba Medical, Tokyo, Japan). All CT examinations included quadruple-phase (unenhanced phase, arterial phase, portal venous phase and equilibrium phase) covering the upper abdomen. Scan parameters: tube voltage 120 kVp; tube current 400 mA, section thickness slice 1 mm. After a routine unenhanced scan, a contrast-enhanced CT scan was performed. A dose of 1.5 mL/kg of warmed contrast medium (Ultravist, Bayer, Germany) was administrated at a rate of 3  mL/s through a 20-G catheter into an antecubital vein, using a mechanical power injector (P3T abdomen module, Medrad Inc.). Hepatic arterial phase, portal venous phase, and equilibrium phase images were obtained at 35 s, 65 s and 180 s, respectively from the start of the contrast material injection.

*MRI examination*

MRI was performed using a 3.0T MR system (Discovery MR750, GE Healthcare) or 1.5T (Optima MR360, GE Healthcare) in a supine position with an eight-channel torso phased-array coil centered over the liver. Scan extent: scanning of the whole liver, from

the top to the lower edge of the liver. Routine MRI sequences included were as follows: T2-weighted, diffusion-weighted, in- and out-of-phase, unenhanced T1-weighted, dynamic, and hepatobiliary phase sequences. The contrast agent was injected into an antecubital vein using an automatic injecting device at a rate of 1.0 mL/s for gadoxetate disodium (Primovist; Bayer Healthcare) for a total dose of 0.025 mmol/kg body weight, or at a rate of 2 ml/s for a dose of 0.1 mmol/kg for the extracellular gadolinium-based contrast agent, followed by a 20 mL saline flush. According to CT/MRI LI-RADS, The timing for the arterial phase was 15–30 s after the contrast agent arrived at the pulmonary artery, with the portal venous phase, transitional phase and hepatobiliary phase at 50–80 s, 3 min and 20 min, respectively.

**The specific process of Focal liver lesion puncture biopsy procedure**

Routine laboratory tests were performed including platelet count, INR, and hemoglobin (Hb) concentration within 1 week before the biopsy. platelet count of ≥50,000/mm^3^ and INR of ≤1.5 are used as a safety reference in our institution. Anti-coagulant and anti-platelet medications were stoped at least 7 days before biopsy. In addition, patients with a history of treatment with anti-platelet or anti-coagulant medications should be monitored for relevant laboratory tests before biopsy. And a comprehensive ultrasound examination should be performed before taking a biopsy to exclude any contraindications.

Biopsy was performed after written informed consent was obtained. All biopsies were performed in the aseptic condition. The patient is placed supine or left supine position, raising his right arm above head. Biopsies were performed via an intercostal or subxiphoid approach. Local anesthesia was administered with 5ml 5% lidocaine subcutaneous injection, then using an 18G Automated Cutting Needle (Bard Biopsy Systems, Tempe, AZ, USA) of 15- or 22-mm core length to achieve the biopsy. Real-time ultrasound guided biopsy in whole process, monitoring the needle path and avoiding intrahepatic vessels, bile ducts and intestines near the liver. One to three punctures are usually performed in our institution. The specimens were fixed in 10% formalin and sent to the pathology laboratory immediately. The biopsy point was scanned through both grayscale and color Doppler mode to check for any post biopsy bleeding, and compressed it about 5 minutes. The patient was sent back to the ward if there was no sign of bleeding. If post-biopsy bleeding was detected, hemostatic measures should be taken immediately to prevent further bleeding, and radiological intervention or surgery may be required when bleeding is severe.

**Table S1: FLLs with TIV images characteristics**

|  | FLL with TIV (n=273) | |
| --- | --- | --- |
|  | HCC (n=266) | non-HCC malignancy (n=7) |
| **Location** |  |  |
| Left liver | 71 (26.7) | 6 (85.7) |
| Right liver | 186 (69.9) | 1 (14.3) |
| Left and right liver | 9 (3.4) | 0 |
| **Size** |  |  |
| <1.9 cm | 2 (0.8) | 0 |
| 2.0-9.9 cm | 153 (57.5) | 4 (57.1) |
| ≥ 10.0 cm | 111 (41.7) | 3 (42.9) |
| **Involved veins** |  |  |
| Main / branch portal vein | 253 (95.1) | 7 (100.0) |
| Inferior vena cava / Hepatic vein | 5 (1.9) | 0 |
| Multiple veins | 8 (3.0) | 0 |
| **US features** |  |  |
| Echogenicity |  |  |
| Hypoechogenicity | 49 (18.4) | 2 (28.6) |
| Isoechogenicity | 2 (0.8) | 0 |
| Hyperechogenicity | 88 (33.1) | 1 (14.3) |
| Mixed echogenicity | 127 (47.7) | 4 (57.1) |
| Margin |  |  |
| Distinct | 109 (41.0) | 4 (57.1) |
| Indistinct | 157 (59.0) | 3 (42.9) |
| Shape |  |  |
| Regular | 135 (50.8) | 4 (57.1) |
| Irregular | 131 (49.3) | 3 (42.9) |
| **CEUS features** |  |  |
| AP enhancement level |  |  |
| Hypoenhancement | 2 (0.8) | 0 |
| Isoenhancement | 8 (3.0) | 1 (14.3) |
| Hyperenhancement | 256 (96.2) | 6 (85.7) |
| Enhancement pattern |  |  |
| Homogeneous APHE | 73 (27.4) | 2 (28.6) |
| Heterogeneous APHE | 192 (72.2) | 4 (57.1) |
| Rim APHE | 1 (0.4) | 1 (14.3) |
| Marked Washout |  |  |
| Yes | 19 (7.1) | 4 (57.1) |
| No | 247 (92.9) | 3 (42.9) |
| Onset time (s) | 13.5 ± 4.2^*^ | 16.6 ± 3.4^*^ |
| Washout time (s) |  |  |
| <60s | 90 (33.8) | 5 (71.4) |
| ≥60s | 176 (66.2) | 2 (28.6) |

Except where indicated, data are numbers of nodules or TIV cases and numbers in parentheses are percentages. * datas are Mean ± SD. **/** means NA.

AP: Arterial phase; APHE: Arterial phase hyperenhancement; CEUS: Contrast-enhanced ultrasound; FLLs: Focal liver lesions; HCC: Hepatocellular Carcinoma; TIV: Tumor in vein; US: Ultrasound
